# Supplementary material for: Double helical conformation and extreme rigidity in a rodlike polyelectrolyte
Source: Nat Commun. 2019 Feb 18;10:801. doi: 10.1038/s41467-019-08756-3 (PMC6379425; doi:10.1038/s41467-019-08756-3)
Supplement: Supplementary file 1 — Supplementary Information [file 41467_2019_8756_MOESM1_ESM.pdf]

Supplementary Information

for

Double-Stranded Helical Conformation and Extreme Rigidity in a Rodlike Polyelectrolyte

Wang et al.

Supplementary Note 1: Persistence length (axial rigidity) of PBDT double helix rods

Based on theories developed by Onsager[1] and Flory[2] that describe the formation of nematic liquid crystalline phases by rodlike particles (which were concisely summarized by Samulski[3]), we can extract the aspect ratio *D/L* of the rodlike particles and thus the axial rigidity persistence length $\underline{L}_p$ from the critical volume fraction of rods $\phi_{nematic}$ at which a nematic phase forms.

Onsager used an athermal model of rods to derive Supplementary Equation 1.[1]

$$\emptyset_{\text{nematic}} \cong 4.5\frac{D}{L} \quad (1)$$

in which *D* is the diameter and *L* is the length of the rods.

In our earlier study of PBDT in solution,[4] on PBDT with a measured M_w = 17,300, we found that the weight fraction at which the nematic phase forms was 0.015 (1.5 wt%). Since the bulk polymer has a density of 1.3 g cm^{-3}, we derive that the volume fraction of polymer will be even lower than the mass fraction. The aspect ratio *D*/*L* would be 300. If we assume that the effective diameter of our double helix rods is 0.8 nm, as given by our XRD studies (the present paper and our previous paper[5]), then the length of the rods will be > 240 nm. This then represents the most conservative value of the axial rigidity persistence length ($L = L_p$) of PBDT.

Flory later used a lattice model to derive Supplementary Equation 2.[2]

$$\emptyset_{\text{nematic}} \cong 12.5\frac{D}{L} \quad (2)$$

In this case, the aspect ratio of the rods would be larger (> 830) and the corresponding persistence length is > 670 nm.

With the higher weight average molecular weight M_w ~ 100 kg mol^{-1} PBDT that we have synthesized recently (see also below), we have observed that the nematic phase forms at a concentration of 0.3 wt%. This polymer thus yields a persistence length of > 1200 nm based on Onsager theory and > 3300 nm from Flory theory. These findings suggest that the persistence length is at least as long as the contour length of the individual polymer chains, and further suggests that the L_p for these double helical structures may be significantly longer than the values reported here. Indeed, L_p may encompass multiple individual polymer chains that entwine (or interleave) axially to form longer double helices than can be achieved with a simple combination of two chains.

Here we note that molecular weight determination for rigid and charged polymers such as PBDT is rarely reliable (using any available method), and so we are vigorously searching for new ways to improve such measurements. As a point of additional information and comparison with our original M_w determination for PBDT stated above,[4] we recently determined the "absolute" molecular weight using a Wyatt MiniDawn LS detector to be 78 kg mol^{-1} (with R_z = 34 nm) for our original polymers, and we observed an apparent 180 kg mol^{-1} (R_z = 60 nm) for the higher molecular weight polymers that show a isotropic-nematic transition at 0.3 wt% in water, where R_z is the length of the polymer chain. At this point, we cannot be certain of any absolute numbers based on the difficulties in determining molecular weights for highly rigid and charged polymers, as mentioned above.

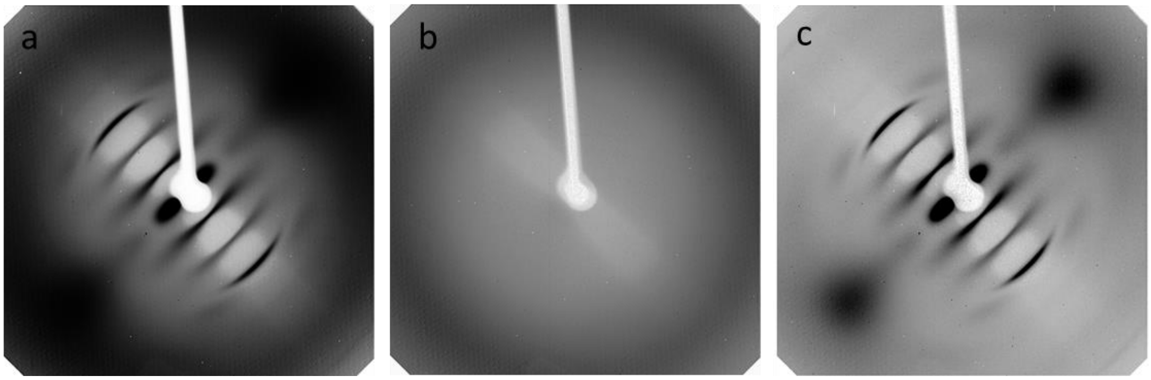

Supplementary Figure 1: (a) X-ray diffraction pattern for 20 wt% PBDT aqueous solution. (b) X-ray diffraction pattern for H_2O. (c) Subtracted diffraction pattern with scale factor α = 0.6. The subtracted figure = (Figure a) – (α × Figure b). (Matlab®)

Supplementary Note 2: Simulation of X-ray diffraction pattern

We have employed the "HELIX" simulation package: Version 19-4-04. The "HELIX" package is freely available from (http://www.ccp13.ac.uk). It is developed by Carlo Knupp and John M. Squire. The detailed information about this software is also available in the software package.

The simulation parameters used in this paper are listed and shown in the HELIX parameter setting interface as displayed in Supplementary Figure 2.

- The axial separation of subunits (h) = 5.6 Å;
- The rotation angle between subunits (β) = 60°;
- The monomer centre radial position (a) = 4 Å;
- The subunit size = 2 Å;
- The azimuthal shift of second strand = 180°;
- The pitch length = h* 360/β = 33.6 Å;

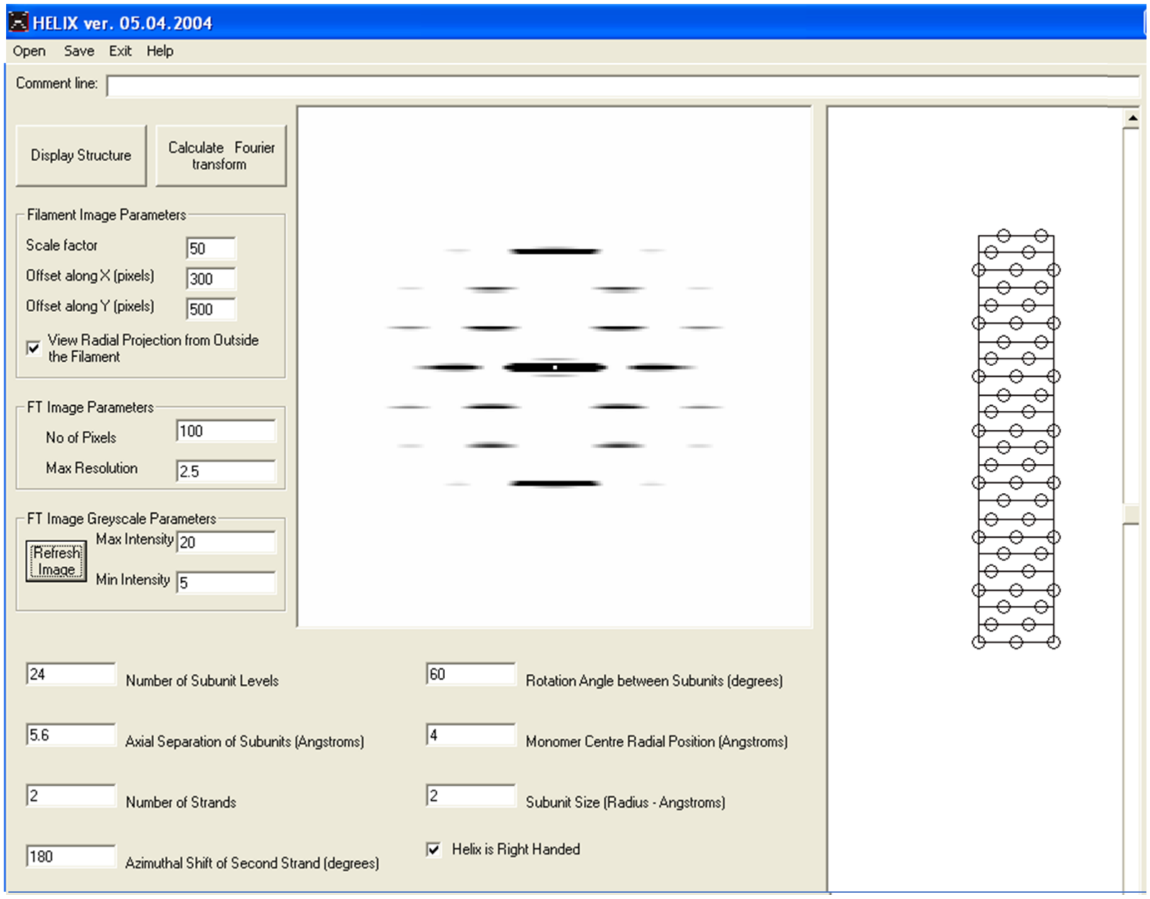

Supplementary Figure 2: The simulation parameters used in the HELIX to get the simulated X-ray diffraction pattern.

Supplementary Note 3: Rod-rod distance

According to our previous study[4], we introduced a hexagonal lattice model for PBDT aqueous solutions.[4] We employed a two-parameter least square regression to find the relationship between the rigid rod-rod distance (*r*) and the weight percentage (*C*) of PBDT polymer solutions.

$$r\ (nm) = \mathrm{A} * C^{-0.5} + \mathrm{B} \tag{3}$$

The fitted result is shown as follows with A = 1.0, B = 0.96 and mean square error = 0.078 in Supplementary Equation 4. Compared to the fitting results in the previous study,[4] the equation is updated based on our new SAXS results at higher concentration, including C = 15 wt% and 20 wt%. We have used the procedure defined in supplementary reference 4, and have simply used these new fitted A and B values in our analysis of the ^{23}Na NMR data.

$$r\ (nm) = 1.0 * C^{-0.5} + 0.96 \tag{4}$$

Supplementary Note 4: NMR spin relaxation and Na^+ dynamics (T_1 and T_2)

Supplementary Figure 3 shows the longitudinal (T_1) and transverse (T_2) spin relaxation times for Na^+ counterions in PBDT polymer solutions. As reported previously by Bull et al., the equality of T_1 and T_2 for ^{23}Na in ionic polymer (polyion) solutions originates from fast motions, such as fast internal rotations and librations, counterion exchange, change of direction of the polyion segments, and counterion diffusion along a polyion rod.[6] Similarly, we have not found any apparent difference between T_1 and T_2 relaxation for ^{23}Na in PBDT solutions. Thus, we demonstrate that there is fast Na^+ motion (fast exchange) along a rod or between rods on at least the ^{23}Na quadrupolar splitting timescale (< 1 s). This demonstration lends further support to the proposed model, where there exists a balance, and averaging, between inter-helical and intra-helical Na^+ exchanges.[4,5]

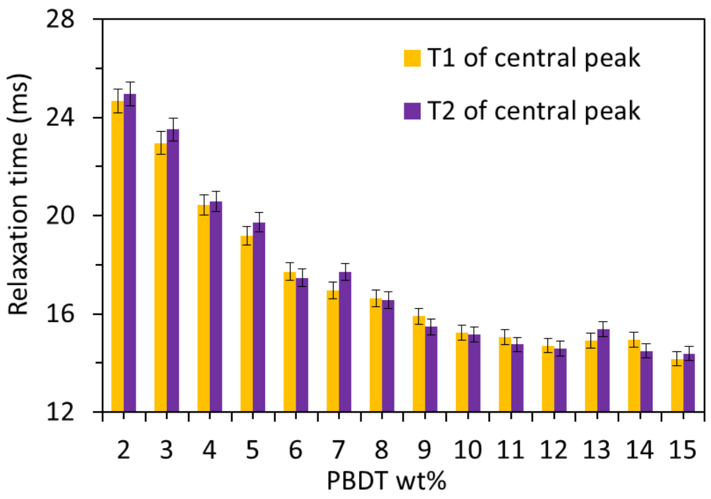

Supplementary Figure 3: T_1 and T_2 spin relaxation of Na^+ counterions in PBDT aqueous solutions as function of the polymer weight percentage in the PBDT polymer solution. The error bars are based on averaging at least three successive measurements of representative relaxation times using this range of experimental parameters and finding the standard deviation, which in this case gives error bars of ±2% of the measured value. Source data are provided as a Source Data File.

Supplementary Note 5: MD simulation equilibrations and double helix self-assembly in water.

We performed two analyses to confirm that the double helical structure is stable and equilibrium was reached in our simulations.

First, we calculated the root-mean-square deviation (RMSD) of the double helical structure of PBDTs using that at ~ 30 ns as a reference structure (the double helical structure was formed by t = 30 ns). Supplementary Figure 4 below shows that the RMSD

is stable and oscillates around ~ 0.25 nm in a narrow range. The magnitude of the oscillation is similar to prior reports of DNA self-assembly in water.[7]

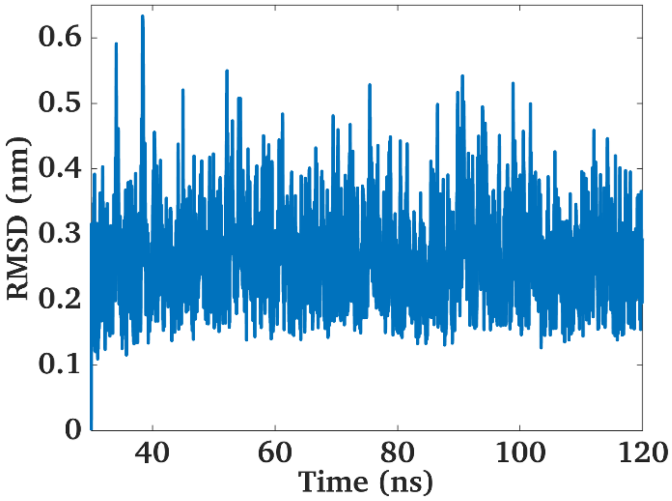

Supplementary Figure 4: The RMSD of the structure formed by the two PBDT monomers in the NPT simulations. The double helical structure was formed by 30 ns, and the structure at this time is taken as the reference structure. Source data are provided as a Source Data File.

Second, for the self-assembled double helix structure, we compute the distance between the SO_3^- groups at the two ends of the rod-like structure as function of time. This distance shows little drift over a time period of 90 ns (see Supplementary Figure 5), thus supporting that the assembled structure has reached equilibrium.

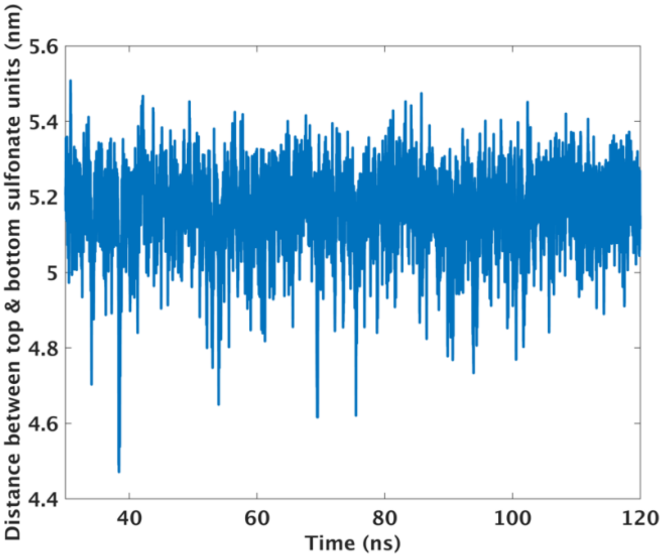

Supplementary figure 5: The distance between the SO_3^- groups at the two ends of the self-assembled double helical PBDT structure in the NPT simulation system during the last 90 ns of the 120 ns-long simulation. The double helical structure was formed by 30 ns. Source data are provided as a Source Data File.

We computed the distribution of the Na^+ counterions around the sulfonate groups in the PBDT. The results are shown in the Supplementary Figure 6a. As a reference, the distribution of water molecules around the sulfonate group is also shown in Supplementary Figure 6b.

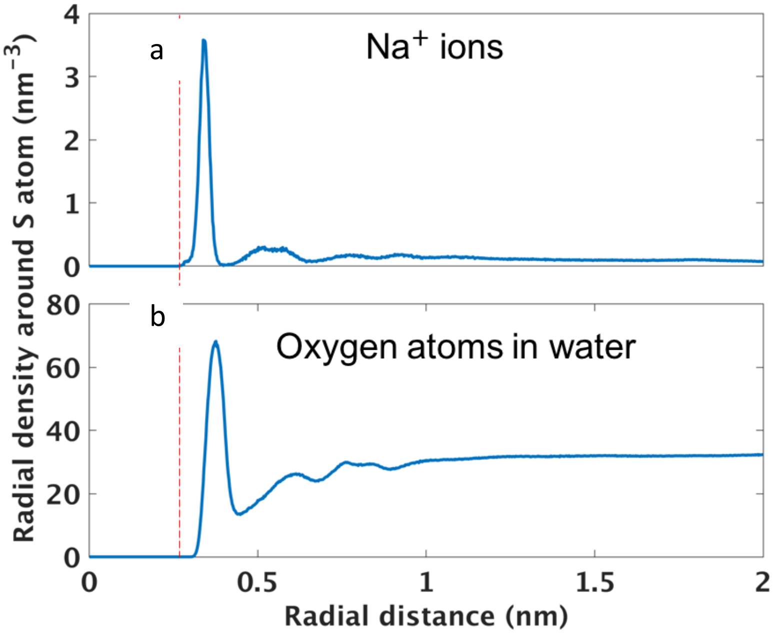

Supplementary Figure 6. The density distribution of Na^+ ions (**a**) and water molecules (**b**) around the sulfur atoms of PBDT's sulfonate groups. These densities are based on the last 90 ns of the NPT simulation. The position of water molecules is based on their oxygen atom. Source data are provided as a Source Data File.

Finally, we mention that two simulations were performed in water with the OPLS-AA force field (NVT ensemble) and with the AMBER force field (NPT ensemble). In both of these other cases, double helices also formed for PBDT as described in the main paper.

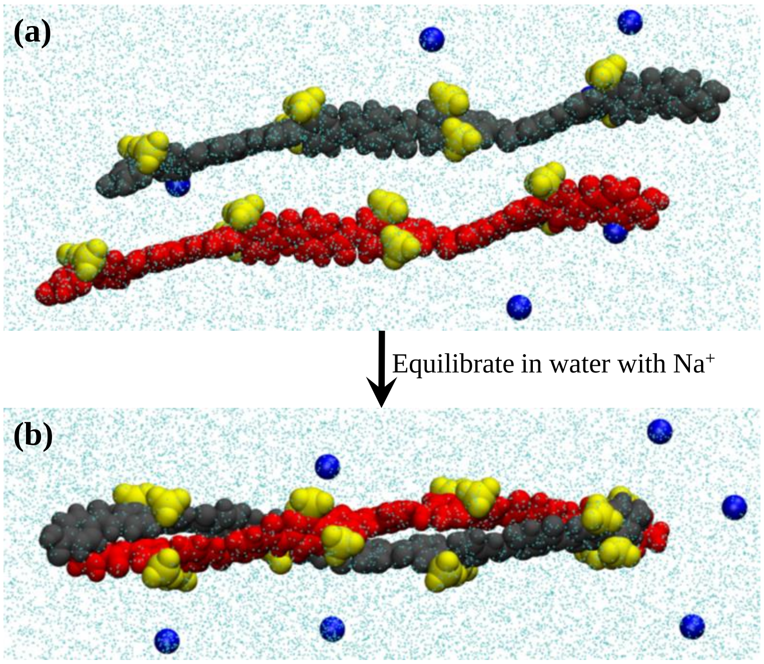

Supplementary Figure 7: Self-assembly of PBDT monomers into a double helix structure. (a) Two PBDT monomers are initially packed side-to-side in water with Na^+ ions. (b) After ≈ 30 ns, the two monomers intertwine with each other to form a double helix structure. The red and black balls denote the backbone of the two PBDT monomers and the yellow balls denote the sulfonate groups. The cyan dots and the blue balls denote the water molecules and the Na^+ ions, respectively. Only a portion of the simulation box and the water/ions in the box are shown for clarity.

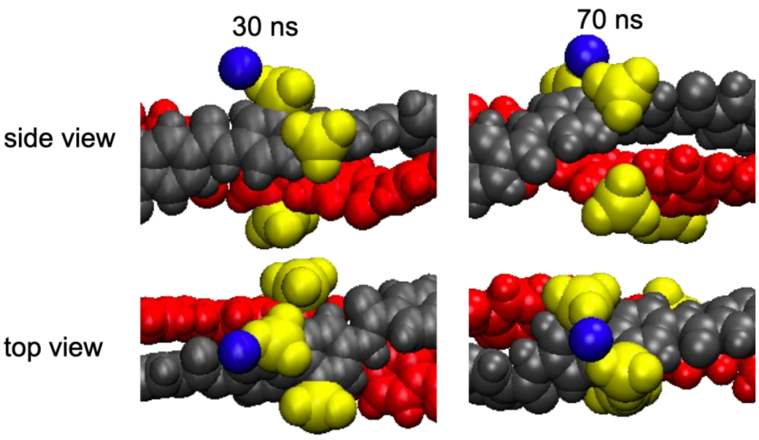

Supplementary Figure 8: Snapshots at 30 ns and 70 ns of two representative frames (top and side view) used when calculating the radial density of Na^+ ions around the sulfur atoms of the PBDT sulfonate groups shown in Supplementary Figure 6. Blue balls denote Na^+ ions, which in these snapshots are located at approximately the most probable radial distance from the $-SO_3^-$ anions (first peak in Supplementary Figure 6).

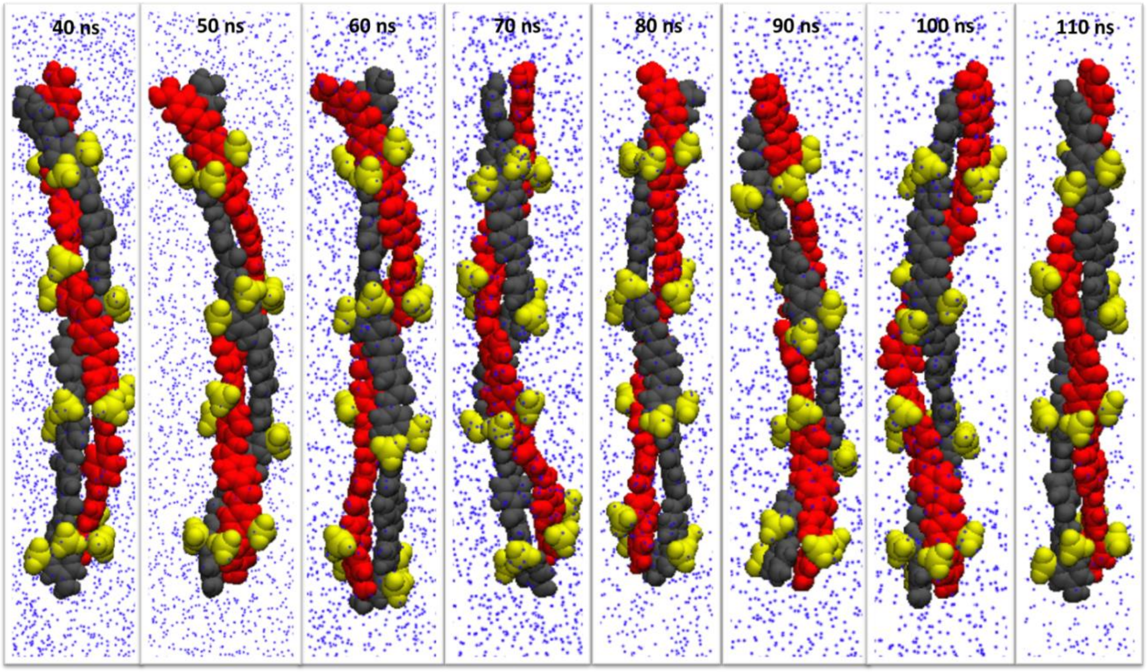

Supplementary Figure 9: Simulation result for two oligomers with 4 monomers in water at T = 300 K with simulation times of 40 ns, 50 ns, 60 ns , 70 ns , 80 ns, 90 ns, 100 ns, and 110ns.

Supplementary Note 6: Initial simulations in vacuum.

Another set of simulations were done in the NVT ensemble (constant temperature and volume) under vacuum conditions as an initial pilot study on this material. We first obtain the optimized structure of the PBDT monomer shown in Supplementary Figure 10a. Starting with polymer chains three monomer units long, we ran simulations lasting 100 ps for two and three chains, with the backbones initially placed at 12 Å distance from each other at $t = 0$. Multiple configurations for these polymers are initially observed in these gas phase simulations, but substantially before 100 ps, we observe only a crossed hydrogen bonding formed between the amide and sulfonate group(Supplementary Figure 10b) and a self-assembled double helix (Supplementary Figure 10c, d). Building on Supplementary Figure 10e, f, Supplementary Figure 11 shows that when three chains are placed in the simulation box, the third chain drifts around the box and does not assemble with the other two chains that form the double helix.

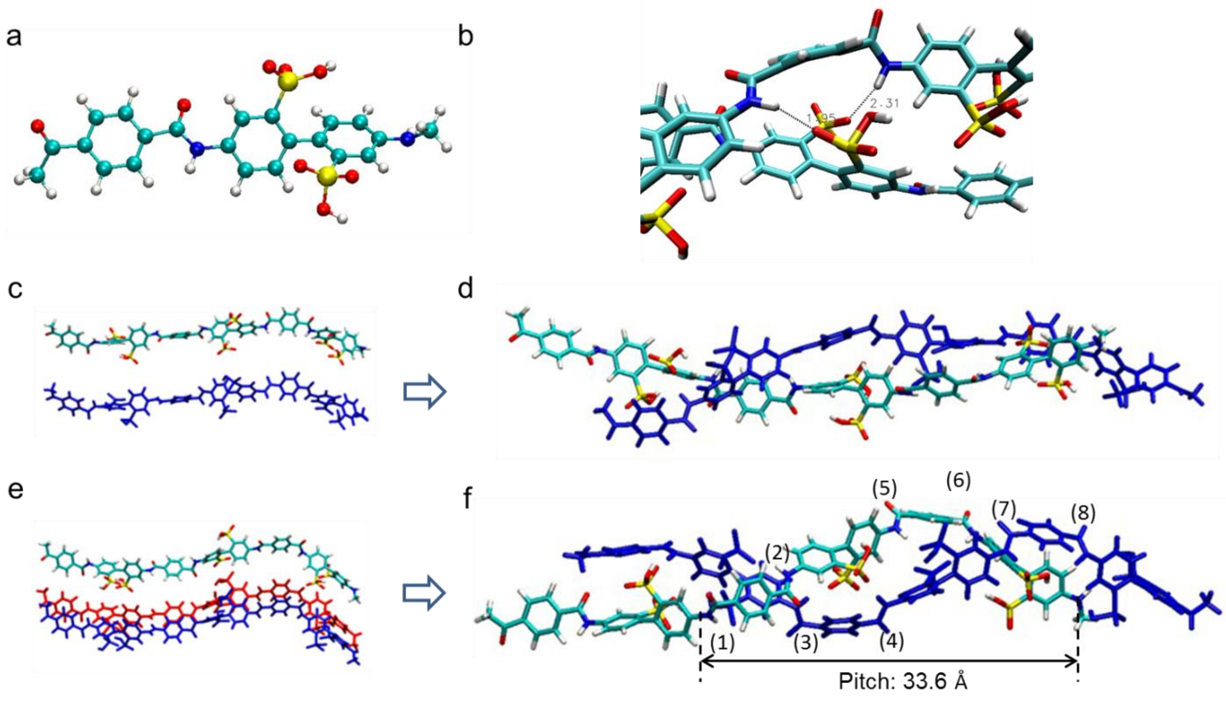

Supplementary Figure 10: (a) The optimized monomer structure of the PBDT in vacuum. (b) Crossed hydrogen bonding formed between the amide and sulfonate group. (c, d) The simulation result for two oligomers in 100 ps in vacuum at T = 300 K. (e, f) The simulation result for three oligomers in 100 ps in vacuum at T = 300 K.

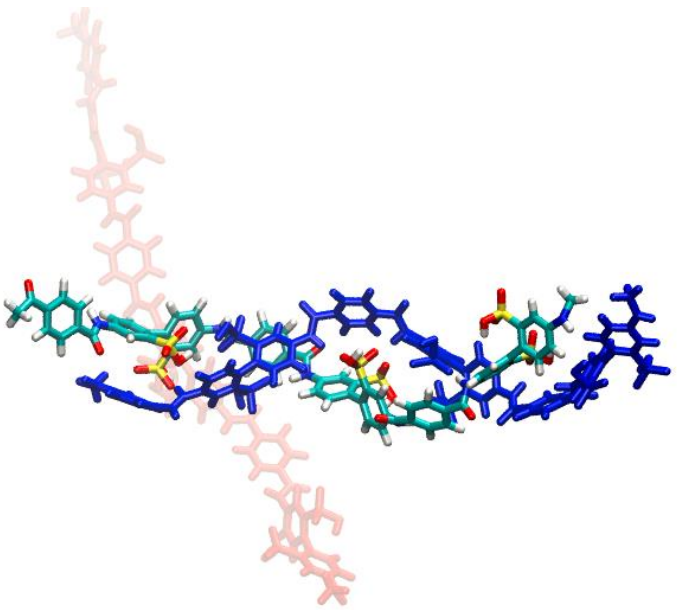

Supplementary Figure 11: The simulation results with three chains in the system. Two chains form a double helix and the third chain drifts in the background.

Supplementary Methods: Simulations of the polymer chains in vacuum were all accomplished with the CM3D molecular dynamics program.[8,9] The force field was the Assisted Model Building with Energy Refinement (AMBER),[10] which is often used for simulating proteins and DNA. Periodic boundary conditions (PBC) were used and a Nosé-

Hoover thermostat at 300 K was used for simulations at a constant temperature. For each simulation at different temperatures, including 10 K, 50 K, 150 K, 250 K, 300 K, 400K, 500 K and 600 K, the runtime was at least 100 picoseconds. The double helix structure forms with simulation temperature above 250 K. In addition, we allowed enough simulation time (up to 300 ps) to verify the stability of the formed double helix structure.

Supplementary References

1 Onsager, L. The Effects of Shape on the Interaction of Colloidal Particles. *Annals of the New York Academy of Sciences* **51**, 627-659, doi:10.1111/j.1749-6632.1949.tb27296.x (1949).

2 Flory, P. J. Phase equilibria in solutions of rod-like particles. *Proceedings of the Royal Society of London. Series A. Mathematical and Physical Sciences* **234**, 73-89, doi:10.1098/rspa.1956.0016 (1956).

3 Samulski, E. T. in *Physical Properties of Polymers ACS Professional Reference Book* (ed J. E. Mark) Ch. 5, 201-264 (Cambridge University Press, 1993).

4 Wang, Y., Gao, J., Dingemans, T. J. & Madsen, L. A. Molecular Alignment and Ion Transport in Rigid Rod Polyelectrolyte Solutions. *Macromolecules* **47**, 2984-2992, doi:10.1021/ma500364t (2014).

5 Wang, Y. *et al.* Highly Conductive and Thermally Stable Ion Gels with Tunable Anisotropy and Modulus. *Advanced Materials* **28**, 2571-2578, doi:10.1002/adma.201505183 (2016).

6 Gustavsson, H., Lindman, B. & Bull, T. Na-23 Nuclear Magnetic-Resonance in Polyanion Solution - Correlation Times and Quadrupole Coupling-Constants of Na+ Bound to Poly(Methacrylic Acid). *Journal of the American Chemical Society* **100**, 4655-4661, doi:Doi 10.1021/Ja00483a003 (1978).

7 Li, M. *et al.* Mutations in DNA-Binding Loop of NFAT5 Transcription Factor Produce Unique Outcomes on Protein–DNA Binding and Dynamics. *The Journal of Physical Chemistry B* **117**, 13226-13234, doi:10.1021/jp403310a (2013).

8 Nielsen, S. O., Moore, P. B. & Ensing, B. Adaptive Multiscale Molecular Dynamics of Macromolecular Fluids. *Physical Review Letters* **105**, 237802, doi:10.1103/PhysRevLett.105.237802 (2010).

9 Ensing, B., Nielsen, S. O., Moore, P. B., Klein, M. L. & Parrinello, M. Energy Conservation in Adaptive Hybrid Atomistic/Coarse-Grain Molecular Dynamics. *Journal of Chemical Theory and Computation* **3**, 1100-1105, doi:10.1021/ct600323n (2007).

10 Wang, J., Wolf, R. M., Caldwell, J. W., Kollman, P. A. & Case, D. A. Development and testing of a general amber force field. *Journal of Computational Chemistry* **25**, 1157-1174, doi:doi:10.1002/jcc.20035 (2004).
