# Supplementary figures and images for: Double helical conformation and extreme rigidity in a rodlike polyelectrolyte
Source: Nat Commun. 2019 Feb 18;10:801. doi: 10.1038/s41467-019-08756-3 (PMC6379425; doi:10.1038/s41467-019-08756-3)

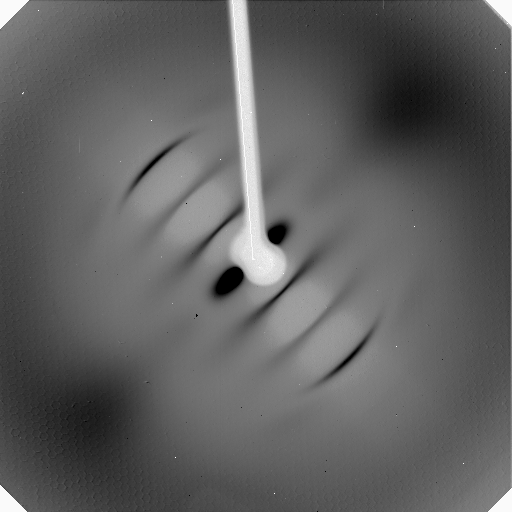

Supplement: Supplementary file 3 — Source Data [file 41467_2019_8756_MOESM3_ESM.zip › 168025_3_data_set_3526426_plhmyh.bmp]

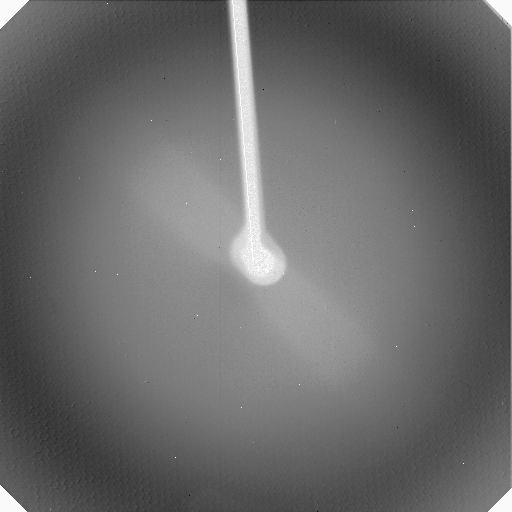

Supplement: Supplementary file 3 — Source Data [file 41467_2019_8756_MOESM3_ESM.zip › 168025_3_data_set_3526427_pl8my8.bmp]

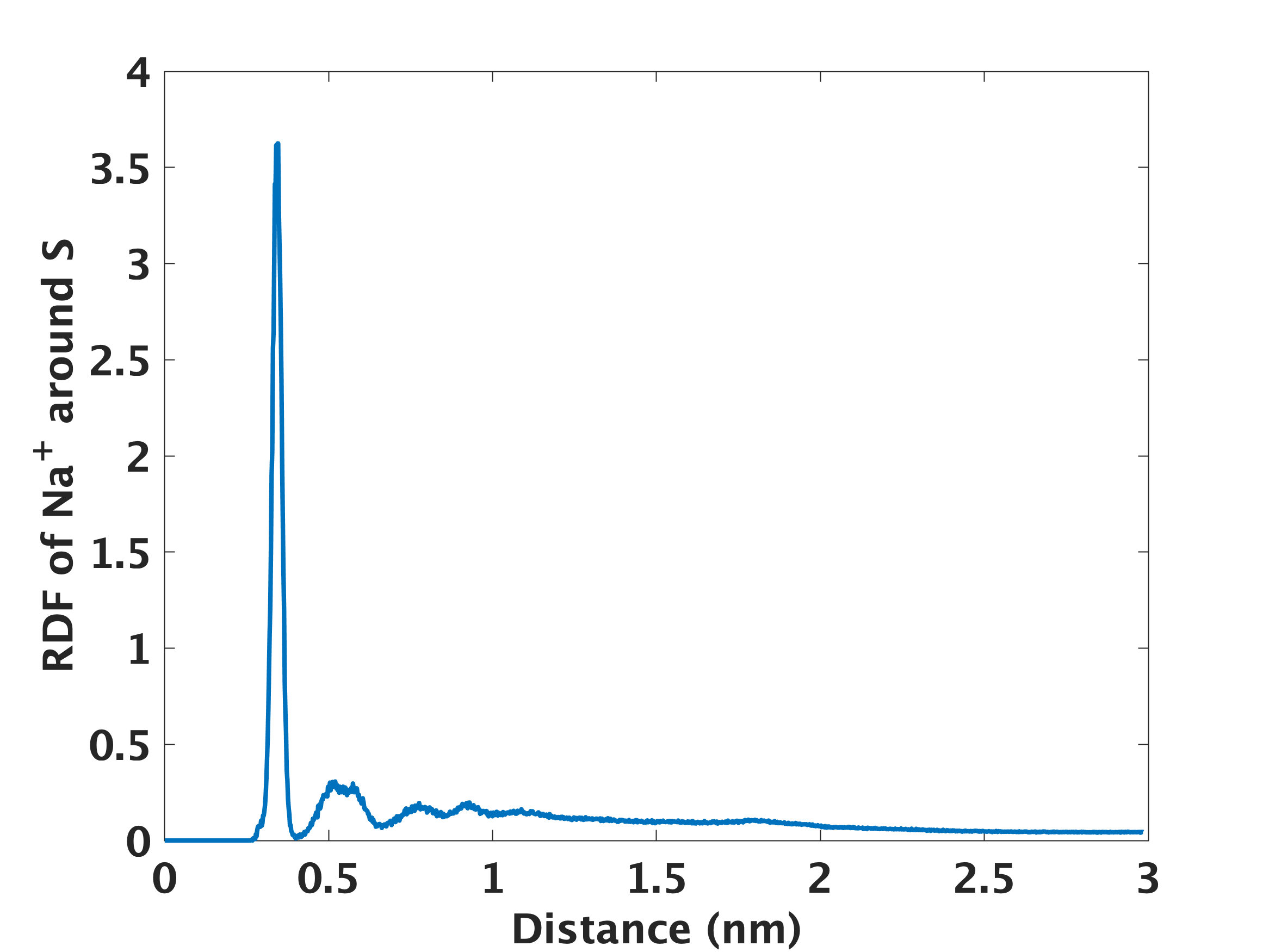

Supplement: Supplementary file 3 — Source Data [file 41467_2019_8756_MOESM3_ESM.zip › 168025_3_data_set_3526435_pl2my2.png]

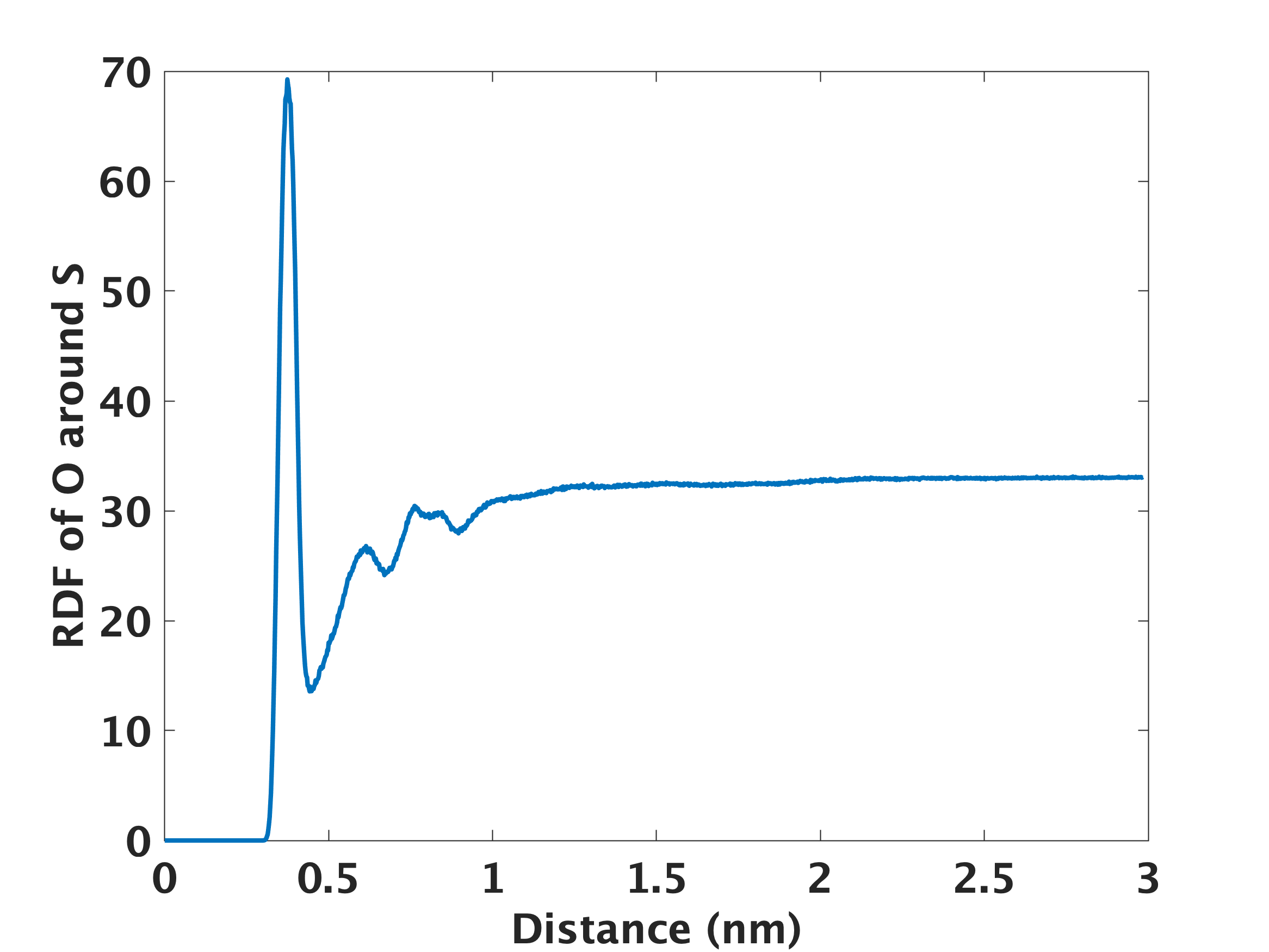

Supplement: Supplementary file 3 — Source Data [file 41467_2019_8756_MOESM3_ESM.zip › 168025_3_data_set_3526436_plhmyh.png]

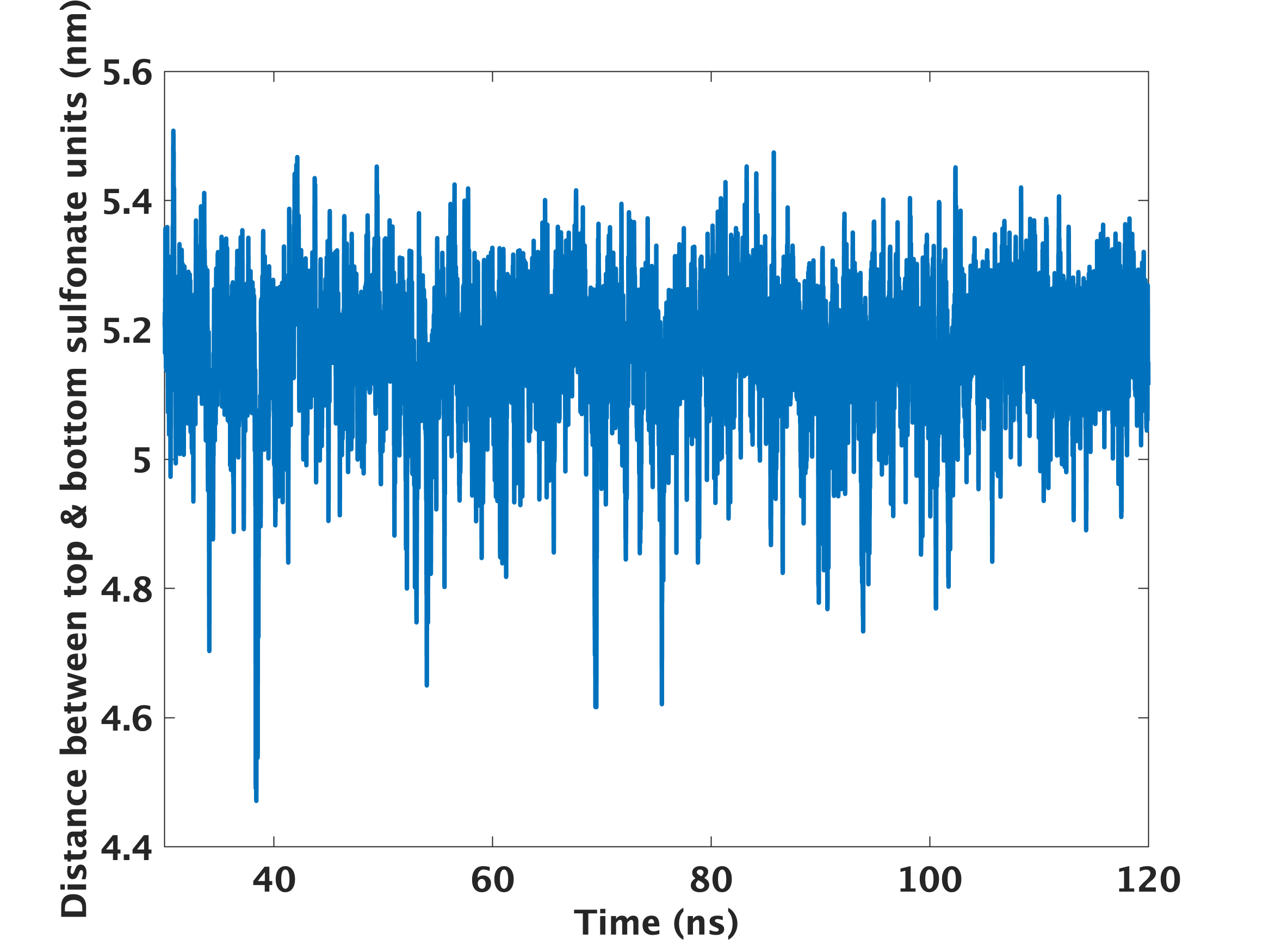

Supplement: Supplementary file 3 — Source Data [file 41467_2019_8756_MOESM3_ESM.zip › 168025_3_data_set_3526429_pl4my4.png]
